# Lysergol monohydrate

**DOI:** 10.1107/S1600536812002632

**Published:** 2012-01-25

**Authors:** Stefan Merkel, Robert Köppen, Matthias Koch, Franziska Emmerling, Irene Nehls

**Affiliations:** a BAM Federal Institute for Materials Research and Testing, Department Analytical Chemistry, Reference Materials, Richard-Willstätter-Strasse 11, D-12489 Berlin-Adlershof, Germany

## Abstract

In the title compound [systematic name: (7-methyl-4,6,6a,7,8,9-hexa­hydro­indolo[4,3,2-*fg*]quinoline-9-yl)methanol monohydrate], C_16_H_18_N_2_O·H_2_O, the non-aromatic ring (ring *C* of the ergoline skeleton) directly fused to the aromatic rings is nearly planar, with a maximum deviation of 0.659 (3) Å, and shows an envelope conformation. In the crystal, hydrogen bonds between the lysergol and water mol­ecules contribute to the formation of layers parallel to (10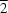).

## Related literature

For the natural occurrence of lysergol, see: Amor-Prats & Harborne (1993 ▶); Uhlig *et al.* (2007 ▶). For the crystal structures of other alkaloids produced by *Clavicipitaceae* see: Pakhomova *et al.* (1995 ▶); Merkel *et al.* (2010 ▶).
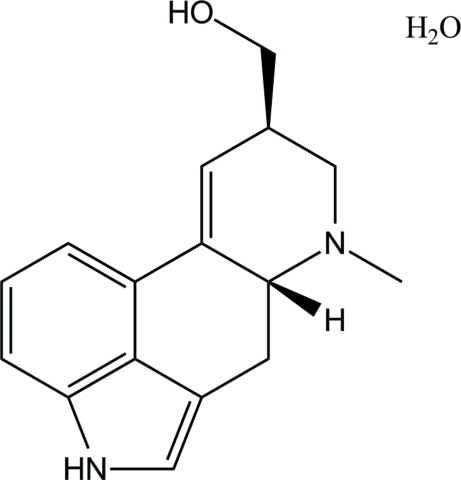


## Experimental

### 

#### Crystal data

C_16_H_18_N_2_O·H_2_O
*M* *_r_* = 272.34
Orthorhombic, 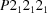

*a* = 7.6234 (12) Å
*b* = 12.3803 (19) Å
*c* = 15.877 (2) Å
*V* = 1498.5 (4) Å^3^
*Z* = 4
Mo *K*α radiation
μ = 0.08 mm^−1^
*T* = 296 K
0.2 × 0.1 × 0.08 mm

#### Data collection

Bruker APEX CCD area-detector diffractometer
Absorption correction: multi-scan (*SADABS*; Bruker, 2001 ▶) *T*_min_ = 0.85, *T*_max_ = 0.96
12705 measured reflections
1569 independent reflections
747 reflections with *I* > 2σ(*I*)
*R*_int_ = 0.122

#### Refinement

*R*[*F* ^2^ > 2σ(*F* ^2^)] = 0.036
*wR*(*F* ^2^) = 0.052
*S* = 0.79
1569 reflections
188 parameters
2 restraints
H atoms treated by a mixture of independent and constrained refinement
Δρ_max_ = 0.10 e Å^−3^
Δρ_min_ = −0.10 e Å^−3^

### 

Data collection: *SMART* (Bruker, 2001 ▶); cell refinement: *SAINT* (Bruker, 2001 ▶); data reduction: *SAINT*; program(s) used to solve structure: *SHELXS97* (Sheldrick, 2008 ▶); program(s) used to refine structure: *SHELXL97* (Sheldrick, 2008 ▶); molecular graphics: *SHELXTL* (Sheldrick, 2008 ▶) and *ORTEPIII* (Burnett & Johnson, 1996 ▶); software used to prepare material for publication: *SHELXTL*.

## Supplementary Material

Crystal structure: contains datablock(s) I, global. DOI: 10.1107/S1600536812002632/fj2506sup1.cif

Structure factors: contains datablock(s) I. DOI: 10.1107/S1600536812002632/fj2506Isup3.hkl

Supplementary material file. DOI: 10.1107/S1600536812002632/fj2506Isup4.mol

Additional supplementary materials:  crystallographic information; 3D view; checkCIF report

## Figures and Tables

**Table 1:** Hydrogen-bond geometry (Å, °)

*D*—H⋯*A*	*D*—H	H⋯*A*	*D*⋯*A*	*D*—H⋯*A*
O1—H1⋯O2^i^	0.82	2.03	2.845 (3)	176
N2—H2*A*⋯O2^ii^	0.86	2.17	2.896 (4)	142
O2—H17⋯N1^iii^	0.84 (3)	2.00 (3)	2.826 (3)	171 (3)
O2—H18⋯O1^iv^	0.84 (3)	1.96 (2)	2.777 (3)	167 (3)

Symmetry codes: (i) 
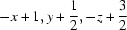
; (ii) 
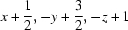
; (iii) 
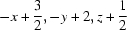
; (iv) 
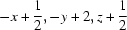
.

**Fig. 1.**
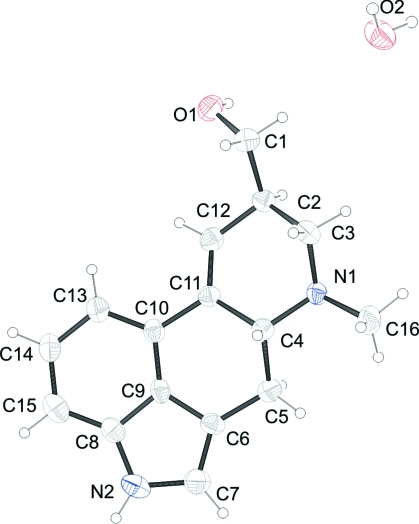
: ORTEP representation of the title compound with atomic labeling shown with 30% probability displacement ellipsoids.

**Fig. 2.**
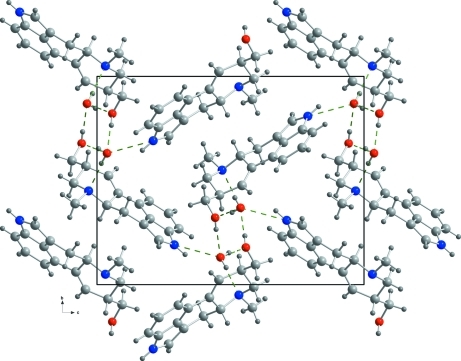
: View of the unit cell of the title compound along [100], showing the hydrogen bonds between the lysergol and adjacent water molecules. Hydrogen bonds are drawn as dashed green lines.

## supplementary crystallographic information

### Comment

Lysergol is a clavine alkaloid produced by fungi of the family *Clavicipitaceae*. It naturally occurs in sclerotia and can be isolated from seeds of some species of the genus *Ipomoea* (Amor-Prats *et al.*, 1993). Lysergol has an ergoline skeleton and is therefore structurally related to ergot alkaloids like ergometrinine (Merkel *et al.*, 2010) or ergotamine (Pakhomova *et al.*, 1995).

The molecule crytsallizes in the orthorhombic space group *P*2_1_2_1_2_1_. The molecular structure of the compound and the atom-labeling scheme are shown in Fig 1.

The absolute configuration could not be defined confidently based on the single-crystal diffraction data. It was however established based on liquid chromatography data that confirmed the epimeric purity of the obtained lysergol crystals. Each lysergol molecule forms four hydrogen bonds to four adjacent water molecules. As a consequence, each water molecule is involved in four hydrogen bonds to four lysergol molecules, resulting in a three dimentional framework structure.

### Experimental

1.4 mg of epimeric pure lysergol (purity > 97%, HPLC-FLD), obtained from Sigma-Aldrich (Taufkirchen, Germany), were dissolved in a glass vial in 1.2 ml of a 84:16 (v:v) acetonitril:water solution. The vial was subsequently capped and stored in the dark at ambient temperature (approximately 23 °C) until crystal formation was complete (2 days). To avoid any epimerization of lysergol to isolysergol the epimeric purity of the resulted crystals was proofed by HPLC-FLD.

### Refinement

In the absence of significant anomalous dispersion effects, Friedel pairs were merged.

The N—H and O—H hydrogen atoms were located in difference maps and and fixed in their found positions (AFIX 3) with Uĩso~(H) = 1.2 of the parent atom U~eq~ or 1.5 *U*~eq~(*C*~methyl~, O).

### Crystal data

**Table:**

C_16_H_18_N_2_O·H_2_O	*F*(000) = 584
*M**_r_* = 272.34	*D*_x_ = 1.207 Mg m^−^^3^
Orthorhombic, *P*2_1_2_1_2_1_	Mo *K*α radiation, λ = 0.71073 Å
Hall symbol: P 2ac 2ab	Cell parameters from 46 reflections
*a* = 7.6234 (12) Å	θ = 4–22°
*b* = 12.3803 (19) Å	µ = 0.08 mm^−^^1^
*c* = 15.877 (2) Å	*T* = 296 K
*V* = 1498.5 (4) Å^3^	Needle, colourless
*Z* = 4	0.2 × 0.1 × 0.08 mm

### Data collection

**Table:**

Bruker APEX CCD area-detector diffractometer	1569 independent reflections
Radiation source: fine-focus sealed tube	747 reflections with *I* > 2σ(*I*)
graphite	*R*_int_ = 0.122
ω/2θ scans	θ_max_ = 25.4°, θ_min_ = 2.1°
Absorption correction: multi-scan (*SADABS*; Bruker, 2001)	*h* = −9→8
*T*_min_ = 0.85, *T*_max_ = 0.96	*k* = −13→14
12705 measured reflections	*l* = −18→18

### Refinement

**Table:**

Refinement on *F*^2^	Primary atom site location: structure-invariant direct methods
Least-squares matrix: full	Secondary atom site location: difference Fourier map
*R*[*F*^2^ > 2σ(*F*^2^)] = 0.036	Hydrogen site location: inferred from neighbouring sites
*wR*(*F*^2^) = 0.052	H atoms treated by a mixture of independent and constrained refinement
*S* = 0.79	*w* = 1/[σ^2^(*F*_o_^2^) + (0.0045*P*)^2^] where *P* = (*F*_o_^2^ + 2*F*_c_^2^)/3
1569 reflections	(Δ/σ)_max_ < 0.001
188 parameters	Δρ_max_ = 0.10 e Å^−^^3^
2 restraints	Δρ_min_ = −0.10 e Å^−^^3^

### Special details

**Table:**

Geometry. All e.s.d.'s (except the e.s.d. in the dihedral angle between two l.s. planes) are estimated using the full covariance matrix. The cell e.s.d.'s are taken into account individually in the estimation of e.s.d.'s in distances, angles and torsion angles; correlations between e.s.d.'s in cell parameters are only used when they are defined by crystal symmetry. An approximate (isotropic) treatment of cell e.s.d.'s is used for estimating e.s.d.'s involving l.s. planes.
Refinement. Refinement of *F*^2^ against ALL reflections. The weighted *R*-factor *wR* and goodness of fit *S* are based on *F*^2^, conventional *R*-factors *R* are based on *F*, with *F* set to zero for negative *F*^2^. The threshold expression of *F*^2^ > σ(*F*^2^) is used only for calculating *R*-factors(gt) *etc*. and is not relevant to the choice of reflections for refinement. *R*-factors based on *F*^2^ are statistically about twice as large as those based on *F*, and *R*- factors based on ALL data will be even larger.

### Fractional atomic coordinates and isotropic or equivalent isotropic displacement parameters (Å^2^)

**Table:**

	*x*	*y*	*z*	*U*_iso_*/*U*_eq_	
O1	0.3895 (3)	1.17847 (16)	0.55746 (12)	0.0635 (7)	
H1	0.4465	1.2344	0.5538	0.095*	
N1	0.9193 (3)	0.95002 (18)	0.53144 (15)	0.0442 (7)	
N2	0.8345 (5)	0.6912 (2)	0.21044 (17)	0.0648 (9)	
H2A	0.8399	0.6452	0.1699	0.078*	
C1	0.4962 (4)	1.0956 (2)	0.5939 (2)	0.0562 (9)	
H1A	0.4266	1.0305	0.6000	0.067*	
H1B	0.5326	1.1182	0.6497	0.067*	
C2	0.6602 (4)	1.0695 (2)	0.5413 (2)	0.0477 (8)	
H11	0.7270	1.1362	0.5326	0.057*	
C3	0.7769 (4)	0.9876 (2)	0.5872 (2)	0.0521 (10)	
H3A	0.8269	1.0210	0.6371	0.062*	
H3B	0.7067	0.9264	0.6051	0.062*	
C4	0.8463 (4)	0.8815 (2)	0.46293 (19)	0.0442 (8)	
H4	0.8020	0.8151	0.4889	0.053*	
C5	0.9912 (4)	0.8488 (2)	0.3992 (2)	0.0571 (10)	
H5A	1.0518	0.9129	0.3795	0.068*	
H5B	1.0762	0.8025	0.4268	0.068*	
C6	0.9116 (5)	0.7897 (3)	0.3247 (2)	0.0488 (9)	
C7	0.9690 (5)	0.7142 (3)	0.2684 (2)	0.0627 (11)	
H7	1.0799	0.6828	0.2684	0.075*	
C8	0.6916 (6)	0.7556 (3)	0.2301 (2)	0.0541 (10)	
C9	0.7374 (4)	0.8162 (3)	0.30186 (19)	0.0458 (9)	
C10	0.6234 (5)	0.8872 (3)	0.34156 (19)	0.0457 (9)	
C11	0.6897 (4)	0.9386 (3)	0.42089 (19)	0.0435 (9)	
C12	0.6124 (4)	1.0234 (2)	0.4570 (2)	0.0514 (9)	
H12	0.5216	1.0566	0.4276	0.062*	
C13	0.4597 (4)	0.9014 (3)	0.30490 (19)	0.0596 (10)	
H13	0.3807	0.9500	0.3284	0.072*	
C14	0.4120 (5)	0.8423 (3)	0.2318 (2)	0.0637 (10)	
H14	0.3014	0.8530	0.2085	0.076*	
C15	0.5253 (6)	0.7691 (3)	0.1937 (2)	0.0678 (12)	
H15	0.4922	0.7305	0.1460	0.081*	
C16	1.0496 (4)	0.8895 (2)	0.58200 (19)	0.0682 (11)	
H16A	0.9984	0.8231	0.6013	0.102*	
H16B	1.0844	0.9322	0.6296	0.102*	
H16C	1.1504	0.8739	0.5479	0.102*	
O2	0.4063 (3)	0.86910 (19)	0.96241 (15)	0.0648 (7)	
H17	0.468 (4)	0.918 (2)	0.9836 (19)	0.097*	
H18	0.327 (3)	0.847 (3)	0.9945 (17)	0.097*	

### Atomic displacement parameters (Å^2^)

**Table:**

	*U*^11^	*U*^22^	*U*^33^	*U*^12^	*U*^13^	*U*^23^
O1	0.0514 (16)	0.0583 (16)	0.0807 (18)	0.0025 (14)	−0.0038 (13)	−0.0068 (13)
N1	0.0467 (19)	0.0461 (17)	0.0398 (17)	0.0035 (15)	−0.0078 (16)	0.0001 (15)
N2	0.090 (3)	0.050 (2)	0.055 (2)	0.003 (2)	0.005 (2)	−0.0091 (16)
C1	0.055 (3)	0.055 (2)	0.058 (2)	0.004 (2)	0.005 (2)	−0.0022 (19)
C2	0.053 (2)	0.044 (2)	0.046 (2)	0.0011 (18)	0.0011 (19)	0.0009 (19)
C3	0.065 (3)	0.047 (2)	0.044 (2)	−0.003 (2)	−0.001 (2)	−0.0040 (18)
C4	0.047 (2)	0.041 (2)	0.045 (2)	−0.0003 (17)	−0.0002 (19)	0.0014 (19)
C5	0.054 (3)	0.059 (2)	0.058 (2)	0.0101 (19)	−0.001 (2)	−0.0029 (19)
C6	0.059 (3)	0.048 (2)	0.040 (2)	0.005 (2)	0.004 (2)	−0.0014 (18)
C7	0.070 (3)	0.063 (3)	0.055 (2)	0.009 (2)	−0.001 (2)	0.001 (2)
C8	0.065 (3)	0.054 (3)	0.043 (2)	−0.006 (2)	0.001 (2)	−0.001 (2)
C9	0.048 (3)	0.055 (3)	0.034 (2)	−0.006 (2)	−0.005 (2)	0.0015 (19)
C10	0.047 (3)	0.053 (2)	0.038 (2)	−0.006 (2)	−0.0012 (19)	0.0022 (18)
C11	0.044 (2)	0.044 (2)	0.043 (2)	0.0015 (18)	0.0029 (18)	0.0008 (18)
C12	0.053 (2)	0.052 (2)	0.049 (2)	0.0046 (19)	−0.001 (2)	0.0075 (18)
C13	0.056 (3)	0.069 (3)	0.053 (3)	0.000 (2)	−0.007 (2)	−0.005 (2)
C14	0.055 (3)	0.075 (3)	0.061 (3)	−0.008 (3)	−0.013 (2)	−0.002 (2)
C15	0.087 (4)	0.067 (3)	0.049 (3)	−0.016 (3)	−0.001 (3)	−0.003 (2)
C16	0.074 (3)	0.066 (3)	0.065 (2)	0.012 (2)	−0.025 (2)	0.002 (2)
O2	0.071 (2)	0.0660 (18)	0.0570 (16)	−0.0169 (14)	0.0090 (14)	−0.0127 (14)

### Geometric parameters (Å, °)

**Table:**

O1—C1	1.432 (3)	C5—H5B	0.9700
O1—H1	0.8200	C6—C7	1.365 (4)
N1—C3	1.476 (3)	C6—C9	1.415 (4)
N1—C16	1.481 (3)	C7—H7	0.9300
N1—C4	1.487 (3)	C8—C15	1.403 (4)
N2—C8	1.385 (4)	C8—C9	1.408 (4)
N2—C7	1.407 (4)	C9—C10	1.388 (4)
N2—H2A	0.8600	C10—C13	1.388 (4)
C1—C2	1.538 (3)	C10—C11	1.499 (4)
C1—H1A	0.9700	C11—C12	1.333 (3)
C1—H1B	0.9700	C12—H12	0.9300
C2—C12	1.500 (4)	C13—C14	1.420 (4)
C2—C3	1.534 (4)	C13—H13	0.9300
C2—H11	0.9800	C14—C15	1.390 (4)
C3—H3A	0.9700	C14—H14	0.9300
C3—H3B	0.9700	C15—H15	0.9300
C4—C11	1.540 (4)	C16—H16A	0.9600
C4—C5	1.552 (4)	C16—H16B	0.9600
C4—H4	0.9800	C16—H16C	0.9600
C5—C6	1.517 (4)	O2—H17	0.838 (10)
C5—H5A	0.9700	O2—H18	0.837 (10)
			
C1—O1—H1	109.5	C7—C6—C9	107.0 (3)
C3—N1—C16	109.1 (2)	C7—C6—C5	135.4 (4)
C3—N1—C4	110.1 (2)	C9—C6—C5	117.6 (3)
C16—N1—C4	111.0 (2)	C6—C7—N2	109.4 (3)
C8—N2—C7	108.0 (3)	C6—C7—H7	125.3
C8—N2—H2A	126.0	N2—C7—H7	125.3
C7—N2—H2A	126.0	N2—C8—C15	133.3 (4)
O1—C1—C2	113.1 (3)	N2—C8—C9	107.1 (3)
O1—C1—H1A	109.0	C15—C8—C9	119.6 (4)
C2—C1—H1A	109.0	C10—C9—C8	123.3 (4)
O1—C1—H1B	109.0	C10—C9—C6	128.2 (3)
C2—C1—H1B	109.0	C8—C9—C6	108.4 (3)
H1A—C1—H1B	107.8	C9—C10—C13	116.9 (3)
C12—C2—C3	108.3 (2)	C9—C10—C11	116.1 (3)
C12—C2—C1	111.5 (3)	C13—C10—C11	127.0 (3)
C3—C2—C1	110.6 (3)	C12—C11—C10	123.1 (3)
C12—C2—H11	108.8	C12—C11—C4	121.2 (3)
C3—C2—H11	108.8	C10—C11—C4	115.5 (3)
C1—C2—H11	108.8	C11—C12—C2	125.1 (3)
N1—C3—C2	110.4 (3)	C11—C12—H12	117.4
N1—C3—H3A	109.6	C2—C12—H12	117.4
C2—C3—H3A	109.6	C10—C13—C14	120.5 (3)
N1—C3—H3B	109.6	C10—C13—H13	119.7
C2—C3—H3B	109.6	C14—C13—H13	119.7
H3A—C3—H3B	108.1	C15—C14—C13	122.2 (4)
N1—C4—C11	110.2 (2)	C15—C14—H14	118.9
N1—C4—C5	111.1 (2)	C13—C14—H14	118.9
C11—C4—C5	112.9 (3)	C14—C15—C8	117.4 (4)
N1—C4—H4	107.5	C14—C15—H15	121.3
C11—C4—H4	107.5	C8—C15—H15	121.3
C5—C4—H4	107.5	N1—C16—H16A	109.5
C6—C5—C4	110.5 (3)	N1—C16—H16B	109.5
C6—C5—H5A	109.6	H16A—C16—H16B	109.5
C4—C5—H5A	109.6	N1—C16—H16C	109.5
C6—C5—H5B	109.6	H16A—C16—H16C	109.5
C4—C5—H5B	109.6	H16B—C16—H16C	109.5
H5A—C5—H5B	108.1	H17—O2—H18	114 (3)
			
O1—C1—C2—C12	−64.4 (3)	C7—C6—C9—C8	−0.4 (4)
O1—C1—C2—C3	175.0 (2)	C5—C6—C9—C8	177.9 (3)
C16—N1—C3—C2	168.4 (2)	C8—C9—C10—C13	−3.2 (5)
C4—N1—C3—C2	−69.5 (3)	C6—C9—C10—C13	178.2 (3)
C12—C2—C3—N1	48.7 (3)	C8—C9—C10—C11	175.5 (3)
C1—C2—C3—N1	171.3 (2)	C6—C9—C10—C11	−3.1 (5)
C3—N1—C4—C11	49.4 (3)	C9—C10—C11—C12	166.3 (3)
C16—N1—C4—C11	170.4 (2)	C13—C10—C11—C12	−15.2 (5)
C3—N1—C4—C5	175.3 (2)	C9—C10—C11—C4	−18.4 (4)
C16—N1—C4—C5	−63.8 (3)	C13—C10—C11—C4	160.1 (3)
N1—C4—C5—C6	−173.7 (2)	N1—C4—C11—C12	−14.6 (4)
C11—C4—C5—C6	−49.3 (3)	C5—C4—C11—C12	−139.5 (3)
C4—C5—C6—C7	−152.8 (4)	N1—C4—C11—C10	170.0 (2)
C4—C5—C6—C9	29.4 (4)	C5—C4—C11—C10	45.1 (4)
C9—C6—C7—N2	−0.5 (4)	C10—C11—C12—C2	172.3 (3)
C5—C6—C7—N2	−178.5 (3)	C4—C11—C12—C2	−2.7 (5)
C8—N2—C7—C6	1.3 (4)	C3—C2—C12—C11	−14.0 (4)
C7—N2—C8—C15	178.3 (4)	C1—C2—C12—C11	−136.0 (3)
C7—N2—C8—C9	−1.5 (4)	C9—C10—C13—C14	2.0 (5)
N2—C8—C9—C10	−177.6 (3)	C11—C10—C13—C14	−176.5 (3)
C15—C8—C9—C10	2.5 (5)	C10—C13—C14—C15	−0.3 (5)
N2—C8—C9—C6	1.2 (4)	C13—C14—C15—C8	−0.4 (5)
C15—C8—C9—C6	−178.6 (3)	N2—C8—C15—C14	179.6 (3)
C7—C6—C9—C10	178.4 (3)	C9—C8—C15—C14	−0.6 (5)
C5—C6—C9—C10	−3.3 (5)		

### Hydrogen-bond geometry (Å, °)

**Table:**

*D*—H···*A*	*D*—H	H···*A*	*D*···*A*	*D*—H···*A*
O1—H1···O2^i^	0.82	2.03	2.845 (3)	176
N2—H2A···O2^ii^	0.86	2.17	2.896 (4)	142
O2—H17···N1^iii^	0.84 (3)	2.00 (3)	2.826 (3)	171 (3)
O2—H18···O1^iv^	0.84 (3)	1.96 (2)	2.777 (3)	167 (3)

Symmetry codes: (i) −*x*+1, *y*+1/2, −*z*+3/2; (ii) *x*+1/2, −*y*+3/2, −*z*+1; (iii) −*x*+3/2, −*y*+2, *z*+1/2; (iv) −*x*+1/2, −*y*+2, *z*+1/2.
